# Digital twin framework for personalized behavioral health optimization with Q-learning and graph-based planning

**DOI:** 10.3389/frai.2026.1834688

**Published:** 2026-08-18

**Authors:** Ayan Chatterjee, Nurilla Avazov

**Affiliations:** 1 Department of Digital Technology, NILU, Kjeller, Lillestrøm, Norway; 2 Inland School of Business and Social Sciences, University of Inland Norway, Lillehammer, Norway

**Keywords:** behavior health optimization, digital twin, Q-learning, recommendation generation, simulation study

## Abstract

Promoting healthy lifestyle behaviors, including physical activity, sleep, diet, stress management, and healthy habits, requires adaptive systems capable of responding to dynamic changes in human behavior. Sustained behavioral change improves individual wellbeing, reduces disease risk, and contributes to healthier societies. However, developing personalized behavioral intervention systems is challenged by demographic heterogeneity, limited and fragmented datasets, reporting inconsistencies, and scarce high-quality labeled data. Ethical, privacy, and cost constraints further restrict the collection of large-scale longitudinal behavioral data. Consequently, there is a need for robust simulation and synthetic data generation frameworks that enable the development and evaluation of adaptive decision-making systems capable of optimizing personalized behavioral interventions over time. This study presents a digital twin framework integrated with tabular Q-learning for personalized behavioral recommendation under World Health Organization (WHO) lifestyle constraints. The framework combines synthetic behavioral data generation, reinforcement learning, and a TSP-inspired planning mechanism to investigate long-term behavioral adaptation in privacy-preserving simulated environments. The digital twin environment models user adherence variability, misreporting, dropout, and behavioral drift, enabling the evaluation of intervention strategies under realistic conditions. Experimental evaluation on synthetic populations demonstrates that Q-learning achieves competitive reward performance while maintaining favorable computational efficiency and stability compared with heuristic and reinforcement learning baselines. Statistical analysis indicates that reward differences among the evaluated methods are not significant; however, the proposed framework provides a flexible platform for adaptive behavioral recommendation and simulation-based experimentation. Furthermore, a real-time recommendation interface illustrates how simulation knowledge can be translated into actionable behavioral guidance. The proposed framework offers a scalable foundation for future digital health systems, particularly in scenarios where data scarcity, privacy constraints, and personalization requirements limit the use of real-world datasets.

## 1 Introduction

Changing behavior toward a healthier life requires more than static advice; it requires a personalized, adaptive system that provides ongoing feedback (Akker et al., 2014; Chatterjee et al., 2021,a,b; Rabbi et al., 2015). This complexity stems from the dynamic interplay of biological rhythms, psychological motivations, social influences, and environmental factors (Deckers, 2018). However, real-life interventions are limited by cost, time, and risk, especially when optimizing for long-term outcomes or tailoring to individual health conditions (Jacob et al., 2025; Peels et al., 2014; Chatterjee et al., 2021, 2022a,b). To overcome these challenges and to tackle data scarcity and privacy related challenges, simulation becomes an indispensable tool. By leveraging reinforcement learning (RL) (Sutton et al., 1998) within a digital twin environment (Vlaev and Dolan, 2015; Cheng et al., 2024; Chatterjee and Avazov, 2026; Digital twin in industry, 2018; Jones et al., 2020), researchers can build a closed virtual ecosystem that simulates the behavior of a real human in a controlled and responsive environment (Xia et al., 2021). The digital twin can act as an alternate self-evolving through feedback, receiving simulated suggestions, and exhibiting measurable behavioral trajectories (Xia et al., 2021). This simulation environment can enable us to test hypotheses, evaluate strategies, and train decision-making processes before actual deployment. It may enable safe research behavioral interventions to avoid health-risks, quick iterations using multiple virtual agents (e.g., users with different profiles), stress-testing under extreme or unusual scenarios (e.g., dropout, misreporting, habit shocks), and quantitative comparison with benchmark algorithms, such as Q-learning (Clifton and Laber, 2020), SARSA (Wang et al., 2013), greedy (Jungnickel, 1999), and local search algorithms [e.g., hill climbing (HC) (Chinnasamy et al., 2022)]. The simulation environment can ensure that reinforcement learning agents can continuously learn from behavioral patterns, improve their reward models (Yu et al., 2021; Frommeyer et al., 2025), and dynamically adapt to WHO guidelines or other relevant compliance frameworks.

Insights gained from recent research advances highlight the need for a robust, adaptive, and simulation-centric framework for behavioral recommendation. The proposed framework builds on the proven utility of digital twins in capturing dynamically changing health states with high-fidelity (Katsoulakis et al., 2024; Rivera et al., 2019; Francis et al., 2024) and the effectiveness of reinforcement learning in facilitating personalized policy learning under deterministic and uncertain constraints in real-time. Furthermore, This study leverages and repur-poses calibration and control mechanisms traditionally used in manufacturing and pharmacology to enhance the realism of digital twins and improve behavioral consistency as inspired by Zheng et al. (2025). The framework is enhanced with active feedback optimization techniques that improve behavioral data quality and system robustness, and RL-based embedding that collaboratively develop complex reward models and behavioral state representations as inspired by Fang et al. (2024). Similarly, Albers et al. (2022) used reinforcement learning to optimize the timing of information delivery in a smoking cessation campaign. Taken together, these contributions highlight the novelty and necessity of combining behavioral optimization with digital twin simulation, building on structured modeling, adaptive learning, and rigorous benchmarking against heuristic baselines and population-level dynamics.

**Figure 1:**
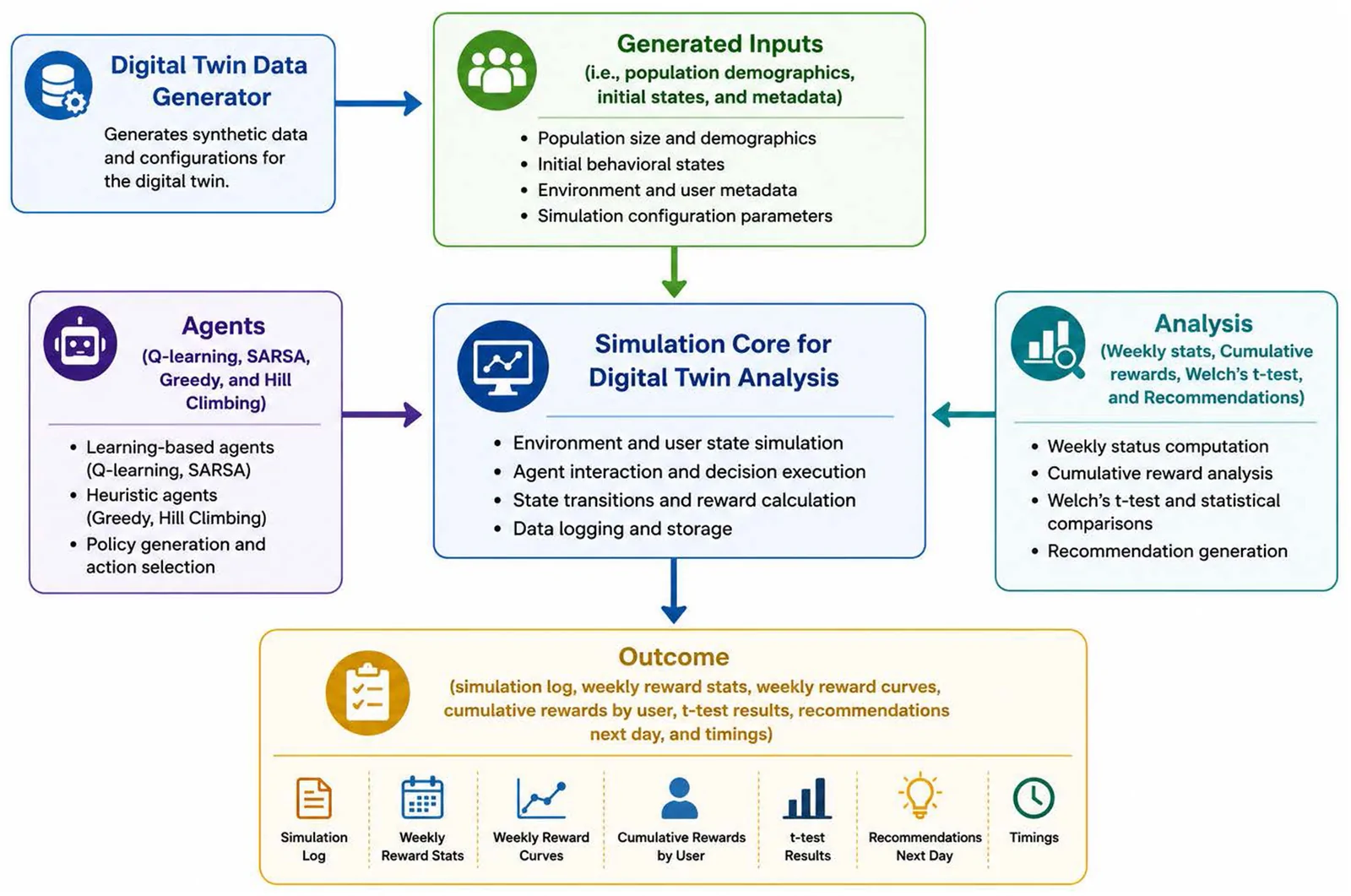
The Data flow diagram in the proposed digital twin system.

This study designs a *digital twin data simulator* that generates population demographics, initial population states, and metadata information, and a *digital twin simulation core* that simulates user's behavior, action states, employ agents for comparative analysis and provides personalized recommendations based on WHO health guidelines. This hybrid simulation and reinforcement learning framework optimizes behavioral trajectories within minimal constraints. Figure 1 illustrates the overall architecture of the proposed digital twin framework. The framework consists of a synthetic data generation layer, a digital twin simulation environment, reinforcement learning agents, evaluation modules, and a real-time recommendation interface. The interaction between these components enables adaptive behavioral optimization under WHO-aligned constraints (World Health Organization Regional Office for Europe, 2025a,b; World Health Organization, 2019, 2020).

To contextualize the contributions of our digital twin framework, this study presents a qualitative comparison with existing methods in Table 1. Adopted approach uniquely integrates constraint programming, real-time behavior simulation, and digital twin fidelity corrections, such as features absent or underexplored in prior research. As shown in Table 1, most previous studies have focused on short-term interventions, lacked formal simulation environments, or failed to integrate optimization formulations to model daily behavior processes. This work addresses these limitations through a unified architecture that combines constraint-based planning, digital twin simulation, reward design under noise, and an on-policy feedback mechanism. This makes proposed framework a scalable, testable, and easily extensible solution for optimizing health behaviors in practice. This simulated approach not only accelerates development but also builds trust and transparency for future integration into clinical or health systems. The main study contributions are summarized as follows—

- Development of a digital twin framework capable of simulating personalized behavioral trajectories under WHO-aligned lifestyle constraints.
- Integration of tabular Q-learning with a constraint-aware behavioral optimization environment to investigate adaptive intervention strategies.
- Introduction of a graph-based planning abstraction, inspired by TSP (Matai et al., 2010), that captures behavioral transition preferences and supports reward shaping for intervention sequencing.
- Design of a synthetic behavioral data generation framework incorporating adherence variability, dropout events, behavioral drift, and misreporting mechanisms.
- Comparative evaluation against reinforcement learning and heuristic baselines under identical simulation settings.
- Demonstration of a real-time recommendation interface that translates simulation-derived policies into actionable behavioral guidance.

**Table 1:** Qualitative comparison of our approach against recent related works.

Feature	Albers et al. (2022)	Fang et al. (2024)	Zheng et al. (2025)	Francis et al. (2024)	This study
RL for behavior change	✓	✓	✓	✓	✓
Digital Twin integration	×	×	✓	✓	✓
Longitudinal simulation	×	×	×	×	✓
Constraint modeling	×	×	×	×	✓
Feedback loop adaptation	×	✓	✓	×	✓
Dropout/realignment scenarios	×	×	×	×	✓
Reward noise handling	×	×	✓	×	✓
WHO compliance alignment	×	×	×	×	✓
Multi-agent simulation support	×	×	×	×	Future Work

The remainder of this paper is organized as follows: Section 2 details our proposed work with problem formulation, Section 3 outlines adopted methodology, Section 3.6 elaborates digital twin framework and its components, study simulation plan, and system description, Section 4 depicts experimental quantitative results and real-time application and Section 5 discusses experimental results with limitations, and future scope. The paper is concluded in Section 6.

## 2 Problem formulation

This study formulates personalized behavioral recommendation as a sequential decision-making problem within a digital twin environment. The objective is to select daily behavioral interventions that improve long-term health-related outcomes while satisfying WHO-aligned lifestyle constraints. The optimization problem aims to maximize cumulative effective reward, defined as the health benefit obtained from behavioral improvements minus penalties associated with constraint violations. The primary mathematical formulation is a Markov Decision Process (MDP) (Garcia and Rachelson, 2013), while the graph-based planning component is used only as an auxiliary reward-shaping mechanism for modeling transition preferences among behavioral interventions.

This study hypothesized that integrating Q-learning with a digital twin framework can optimize behavioral changes [e.g., physical activity, sleep, diet, stress level (ε {0 – 10}), and habits (e.g., tobacco, alcohol)], resulting in higher cumulative rewards and better compliance with WHO guidelines compared to baseline heuristics and non-reinforcement learning methods. Our optimization problem has maximized cumulative health rewards and minimize constraint violations over simulation days. Based on the Bellman's optimality principle (Leibfried et al., 2019) and the framework of constraint programming (Rossi et al., 2008), this study will prove that the Q-learning method will ultimately lead to an optimal or near-optimal behavioral policy.

**Null Hypothesis (***H*_0_**):** Q-learning's mean cumulative reward is equal to other methods. *H*_0_: μQ-Learning = μOthers.

**Alternative Hypothesis (***H*_1_**):** Q-learning's mean cumulative reward is significantly higher than other methods. *H*_1_: μ_Q − Learning_ > μ_Others_.

### 2.1 Behavioral optimization objective

Let *s*_*t*_ ε *S* denote the behavioral state of a user at time *t*, and let *a*_*t*_ ε *A* denote the selected behavioral intervention. After action *a*_*t*_ is applied, the user transitions to a new state *s*__*t*_+1_ and receives an immediate reward *R*_*t*_. Constraint violations are penalized by *L*_*t*_, resulting in the effective reward:


$$\begin{array}{l} r_{t}^{\text{eff}}=R_{t}-L_{t}. \end{array}$$ (1)

The optimization objective is to learn a policy π that maximizes the expected cumulative discounted effective reward over a finite horizon *T* :


$$\begin{array}{l} \underset{\pi }{\max}{𝔼}_{\pi }\left[\overset{T}{\underset{t=0}{\sum }}\gamma^{t}r_{t}^{eff}\right] \end{array}$$ (2)

This formulation explicitly connects the state, action, reward, penalty, and transition dynamics used in the proposed digital twin environment.

#### 2.1.1 Markov decision process (MDP) formulation

Behavioral change is inherently a sequential decision-making problem in which the future health state of an individual depends on both the current state and the intervention applied. Consequently, the problem can be naturally formulated as a Markov Decision Process (MDP). The adopted MDP representation allows the framework to model delayed rewards, uncertainty in adherence, dropout events, and stochastic behavioral transitions. Table 2 depicts a behavioral state representation used by the MDP. Although the current proof-of-concept implementation contains only six intervention categories, the primary motivation for reinforcement learning is not combinatorial complexity but rather the ability to learn adaptive long-term policies under uncertainty. The proposed framework is intended to support future extensions involving larger intervention spaces, intervention timing strategies, and context-aware recommendations where exhaustive search becomes infeasible.

**Table 2:** Behavioral state representation used by the MDP.

State variable	Range	Description
Sleep	0–10	Sleep quality score
Activity	0–10	Physical activity level
Diet	0–10	Dietary adherence score
Hydration	0–10	Water intake compliance
Stress	0–10	Stress index
Habit	0–10	Tobacco/alcohol avoidance score

#### 2.1.2 Graph-inspired planning

The proposed framework does not employ Traveling Salesman Problem (TSP) (Matai et al., 2010) as a strict optimization requirement. Instead, the TSP formulation serves as a planning abstraction that encourages structured intervention sequencing and discourages repetitive recommendations. The objective is not to enforce a classical Hamiltonian tour but to exploit graph-based transition modeling for behavioral planning. The TSP-inspired edge costs are incorporated as a reward-shaping mechanism that captures both transition desirability and transition difficulty between behavioral states.

The TSP (Matai et al., 2010) is a classical combinatorial optimization problem defined on a weighted graph. Let *G* = (*V, E, W*) be a complete graph where *V* = {*v*_1_*,..., v*_*n*_} is a set of nodes (cities), *E* is the set of edges connecting all pairs of nodes, and *W* = {*w*_*ij*_} assigns a cost (distance) to each edge (*i, j*) ε *E*. The goal of TSP is to determine a minimum-cost Hamiltonian cycle, i.e., a tour that visits each node exactly once and returns to the starting node.

Formally, a tour π is an ordering of nodes such that every node is visited once. The cost of a tour is the sum of edge weights along the path:


$$\text{Cost}(\pi )=\sum_{k=1}^{n}w_{\pi {\left(k\right)}_{\pi \left(k+\text{1}\right)}},\;$$ (3)

where π(*n* + 1) = π(1) ensures the tour is closed. The standard TSP objective is:


$$\begin{array}{l} \underset{\pi \in \Omega }{\min}\text{Cost(}\pi \text{),} \end{array}$$ (4)

where Ω denotes the set of all valid Hamiltonian tours on *V*.

TSP can be *symmetric* (STSP) if *w*_*ij*_ = *w*_*ji*_ for all *i, j*, or *asymmetric* (ATSP) if edge costs depend on direction, i.e., *w*_*ij*_ = *w*_*ji*_. Because the number of possible tours grows factorially with *n*, TSP is NP-hard and is commonly solved using exact methods (e.g., integer linear programming, branch-and-bound) or heuristics and metaheuristics (e.g., nearest neighbor, 2-opt/3-opt, genetic algorithms, simulated annealing).

#### 2.1.3 Optimization model

Each health behavior is modeled as a state in a Markov Decision Process (MDP) (Davis, 2018). Actions correspond to transitions between behaviors within a daily plan. The objective is to maximize cumulative health rewards, constrained by WHO recommendations.

**Definitions:**

- *S*: the set of behavioral states (e.g., sleep quality, activity level, diet score, stress index).
- *A*: the set of possible behavioral interventions (e.g., walk 30 min, skip alcohol, sleep early, meditate).
- *R*(*s, a*): reward function encoding health benefit (aligned with WHO guidelines) (e.g., sleep: 2.5, activity: 2.0, diet: 2.0, habit: 3.0, stress: 1.5, hydration: 1.0).
- *Q*(*s, a*): expected cumulative reward of taking action *a* in state *s*.
- γ: discount factor for future behavioral impact.
- α: learning rate for future behavioral impact.
- π^*^: optimal policy.

Given states


$$\begin{array}{l} S=\left\{s_{\text{1}},...,s_{n}\right\}, \end{array}$$ (5)

Q-values *Q*(*s, a*) are learned using the standard Bellman update rule (Q-Learning):


$$\begin{array}{l} Q\left(s,a\right)\leftarrow Q\left(s,a\right)+\alpha \left[R+\gamma \underset{a^{\prime }}{\text{ max}}Q\left(s^{\prime },a^{\prime }\right)-Q\left(s,a\right)\right]. \end{array}$$ (6)

*s* and *a* denote the current state and executed action, while *s*′ is the resulting next state and *a*' ranges over all possible actions in *s* to compute the greedy target ${\text{max}}_{a}\prime$ *Q*(*s, a*).

**Explicit mapping of RL states to the TSP layer:** to express daily sequencing as a TSP, each behavioral state *s*_*i*_ ε *S* is mapped to a graph node *v*_*i*_. Indices *i* and *j* therefore *label nodes* (behaviors) in the graph, not MDP time steps. A directed edge (*i, j*) represents the transition from node *v*_*i*_ to node *v*_*j*_, i.e., from state *s*_*i*_ to state *s*_*j*_.

The context-aware edge cost is defined as:


$$\begin{array}{l} \text{Cost}\left(i,j\right)=\lambda P_{ij}-R_{ij}, \end{array}$$ (7)

where *P*_*ij*_ is the penalty and *R*_*ij*_ is the reward associated with the directed graph transition *v*_*i*_→*v*_*j*_, and λ is a penalty multiplier. Thus, Cost(*i, j*) quantifies how desirable it is to move between two behavioral nodes in the TSP tour, while the RL update governs how action values evolve over time.

Objective:


$$\begin{array}{l} \underset{\pi }{\max}{𝔼}_{\pi }\left[\overset{T}{\underset{t=1}{\sum }}\gamma^{t}R\left(s_{t},\pi \left(s_{t}\right)\right)\right]\backslash n \end{array}$$ (8)

In the above objective, the horizon *T* denotes the finite planning time length (episode duration) over which the cumulative discounted reward is evaluated and optimized. It corresponds to the final time step of a trajectory, so the sum $\overset{T}{\underset{t=0}{\sum }}$considers rewards only from the current step (*t* = 0) up totime *T*. In the digital-twin eCoaching setting, T may represent a day (e.g., *T* = 24 hourly steps), aweek (e.g., *T* = 7 daily steps), or a full program duration (e.g., *T* = 365 days). Therefore, maximizing reward over horizon *T* means selecting a policy π that yields the best expected discounted health outcome within this finite window.

**Daily Constraints for a healthy lifestyle (WHO-aligned World Health Organization Regional Office for Europe**, 2025a**,b; World Health Organization**, **2019**, **2020****):**

Activity: Minutes(activity_*t*_)≥30 Sleep: 7 ≤ hours(sleep_*t*_) ≤ 9 Diet: fruits+veg_*t*_ ≥ 400*g* Habit: no tobacco/alcohol

Hydration: glasses(water_*t*_) ≥ 8

Let:

- *x*_*i, t*_ ε *A* be the action taken on day *t* for user *i*.
- *C*_*j*_(*x*_*i, t*_) be the *j*th constraint function at time *t*.
- *R*(*x*_*i, t*_) be the reward for taking action *x*_*i, t*_.

**Penalty function:**


$$\begin{array}{l} L_{t}=\underset{j}{\sum }\lambda_{j}\cdot 1\left[C_{j}\left(x_{i,t}\right)\text{violated}\right]\text{,} \end{array}$$ (9)

where λ_*j*_ is the penalty weight for constraint *j*, and **1**[·] is the indicator function.

**Total objective:**


$$\begin{array}{l} \text{max}\overset{N}{\underset{t=1}{\sum }}\left(R\left(x_{i,t}\right)-L_{t}\right). \end{array}$$ (10)

Here, *L*_*t*_ denotes the total constraint-violation penalty at time step *t*. It aggregates the penalties from all WHO-aligned constraints, such that each violated constraint *C*_*j*_ adds a weighted cost λ_*j*_ via the indicator function **1**[·]. Therefore, *L*_*t*_ quantifies how much the action *x*_*i, t*_ deviates from the desired healthy lifestyle requirements, and it is subtracted from the reward in the total objective.

#### 2.1.4 Reward function design

The immediate reward is computed as a weighted aggregation of WHO-aligned behavioral indicators:


$$\begin{array}{l} R_{t}=w_{\text{1}}Sleep_{t}+w_{\text{2}}Activity_{t}+w_{\text{3}}Diet_{t}+w_{\text{4}}Hydration_{t}\\ \;+w_{\text{5}}Habit_{t}-w_{\text{6}}Stress_{t} \end{array}$$ (11)

where

(*w*_1_, *w*_2_, *w*_3_, *w*_4_, *w*_5_, *w*_6_) = (2.5, 2.0, 2.0, 1.0, 3.0, 1.5)

Represent empirically selected weights reflecting the relative importance of each behavioral dimension. Constraint violations are incorporated through: ${r_{t}}^{eff}=R_{t}-L_{t},$ which represents the effective reward used by the Q-learning update rule. The selected action modifies one or more behavioral state variables, resulting in a new state from which the immediate reward is calculated. The effective reward is then used to update the Q-table according to the Bellman equation.

#### 2.1.5 Graph-based planning inspired by TSP

The proposed framework does not solve a classical TSP. Instead, it adopts a TSP-inspired graph representation to guide transition-aware reward shaping and behavioral intervention sequencing. This optimization frame, each behavior state (e.g., Activity, Sleep, Diet, Habit) is represented as a “city” (graph node), and directed edges between nodes represent feasible transitions between behaviors (see Table 3). Each edge encodes the cost of moving from one behavior to another, capturing both health benefit and constraint-related effort. The objective is to find an efficient tour that visits all required behavior nodes within a day or week without repetition, maximizing net health gain in the same spirit as minimizing travel cost in classical TSP. The proposed TSP-inspired formulation does not require each intervention to be executed exactly once. Instead, the graph abstraction is used to model transition preferences among intervention categories during planning. Individual interventions may be revisited across episodes, while the planning layer encourages balanced behavioral coverage within a planning horizon.

**Table 3:** Mapping to TSP elements for graph representation of the problem.

Behavioral modeling component	TSP equivalent	Meaning
Health behavior state	Node (City)	A lifestyle component (e.g., Sleep, Diet)
Action sequence	Path (Tour)	Order of behavioral interventions
Transition cost	Edge weight (cost/reward)	Difficulty or ease of switching behaviors
Health benefit	Negative cost/reward	Desirable transitions (e.g., Sleep → Exercise)
Health penalty	Positive cost	Undesirable behavior (e.g., Diet → Tobacco)
Objective	Shortest/optimal tour	Maximum reward/minimum penalty cycle

To make the graph context-sensitive, each directed edge (*i, j*) is assigned a dynamic weight:


$$\begin{array}{l} w_{ij}=-r_{ij}+\lambda \cdot p_{ij}, \end{array}$$ (12)

where *i* and *j* are node indices corresponding to two behavioral states in the TSP graph, and (*i, j*) denotes the directed transition *i*→*j*. The term


$$\begin{array}{l} r_{ij}=\text{reward associated with the transition}i\to j, \end{array}$$ (13)

captures the expected health benefit of shifting from behavior *i* to behavior *j*, while


$$\begin{array}{l} p_{ij}=\text{penalty incurred if the transition}i\\ \;\to j\text{violates soft}/\text{hard constraints}, \end{array}$$ (14)

quantifies deviations from WHO-aligned lifestyle requirements. The scalar


$$\begin{array}{l} \lambda =\text{tunable penalty multiplier}, \end{array}$$ (15)

controls the trade-off between reward maximization and constraint satisfaction.

Edge rewards are defined as follows:


$$\begin{array}{l} \text{rewar}{\text{d}}_{ij}=\text{1}0-\text{cos}{\text{t}}_{ij}. \end{array}$$ (16)

Here, *i* and *j* are node indices in the TSP graph, where each node corresponds to a behavioral state; thus, (*i, j*) denotes a directed transition from behavior *i* to behavior *j*, and *w*_*ij*_, *r*_*ij*_, and *p*_*ij*_ are edge-specific cost, reward, and penalty terms. This formulation promotes transitions consistent with healthy behavior and penalizes unfavorable or unpredictable changes. The Q-table learns action values through *Q*(*s, a*), while the graph-based planning representation encourages balanced and transition-aware behavioral sequencing. This component supports reward shaping but does not replace the MDP as the primary decision-making model. In reward equation, the constant value of 10 has been selected to normalize transition rewards within a bounded positive range, ensuring numerical stability during reinforcement learning updates. This value is an empirical normalization constant rather than a theoretically derived parameter.

### 2.2 Proposed algorithm with complexity analysis

The proposed Algorithm 1 uses Q-learning to optimize daily behavioral choices over a specified time horizon (e.g., 365 days). It balances exploration and exploitation by applying a ϵ-greedy policy while simultaneously updating Q-values to maximize long-term health benefits. The method ensures compliance with WHO guidelines through integrated constraints and penalties for violations.

Algorithm 1 Constraint-aware Q-learning for behavioral optimization. 
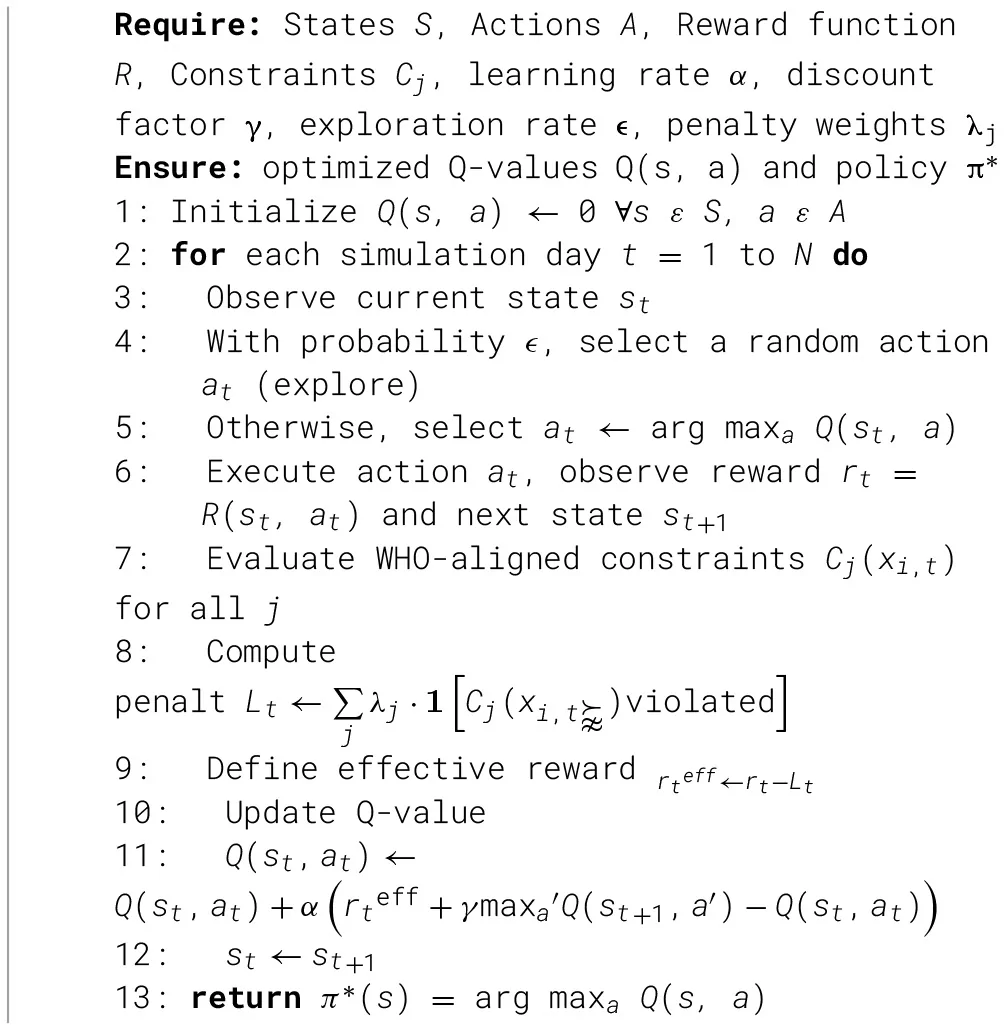

The computational complexity of Algorithm 1 depends on the state and action space sizes. The computational complexity of Algorithm 1 depends on the size of the state and action spaces as well as the evaluation of constraints. Let, |*S*| denotes the number of states and |*A*| denotes the number of possible actions. Each Q-value update requires evaluating all possible actions, resulting in a per-step complexity of O(|*A*|). Moreover, checking constraint violations introduces an overhead proportional to the number of constraints |*C*|, yielding a per-step cost of O(|*A*| + |*C*|). Over a planning horizon of *N* days (episodes), the total computational cost becomes: O *N* · (|*A*| + |*C*|), assuming a sparse Q-table representation. This complexity remains tractable for moderate |*S*|, |*A*|, and |*C*|, making the approach suitable for large-scale digital twin simulations of behavioral health.

## 3 Methodology

**Figure 2:**
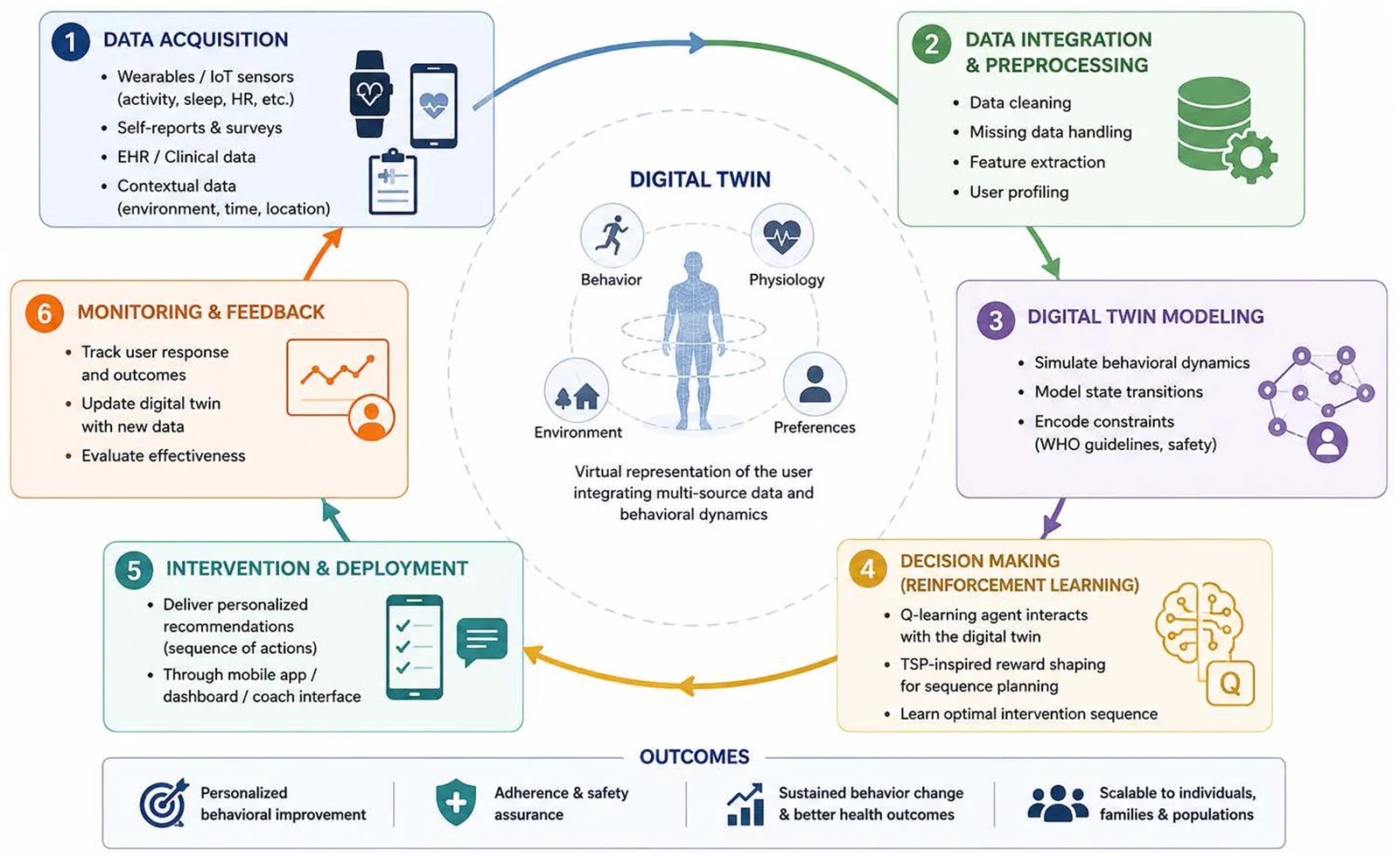
Lifecycle of the proposed digital twin framework for personalized behavioral optimization, illustrating data acquisition, digital twin modeling, reinforcement learning, recommendation generation, and continuous feedback.

The methodology adopted in this study integrates Q-learning, constraint programming, and digital twin simulation to optimize behavioral health interventions. As shown in Figure 2, the proposed framework follows a closed-loop optimization process in which user information is first transformed into a digital twin representation. The reinforcement learning agent interacts with this virtual representation to identify personalized intervention strategies while considering WHO guideline constraints and behavioral transition costs. User responses and behavioral outcomes are continuously monitored and incorporated into subsequent model updates, allowing the digital twin to evolve over time and improve recommendation quality. This lifecycle demonstrates how the proposed framework integrates simulation, optimization, and real-time recommendation generation within a unified digital health ecosystem (Topol, 2019).

### 3.1 Digital twin construction

A population of synthetic users has been generated to represent heterogeneous demographic and behavioral characteristics, including age, gender, BMI, baseline activity, adherence propensity, work schedule, stress level, and smoking status. Each user's daily state encompassed six dimensions—sleep, activity, diet, habits, stress, and hydration. Transitions between states were modeled taking into account stochasticity in adherence, information noise, and weekly dropout/reorganization dynamics to mimic realistic variability.

### 3.2 Reinforcement learning framework

The optimization problem is formulated as a MDP, where states represent health-promoting behaviors and actions correspond to interventions (e.g., “Go to bed early,” “Run for 30 min”). Rewards are adjusted according to WHO guidelines, and penalties are used to catch violations. To address transition difficulties, a shaping term, inspired by TSP, is added to the edge weights between action states. The proposed Q-learning algorithm updates action values according to the Bellman optimality principle, and ablation studies investigate the effects of removing shaping, penalties, or noise. SARSA, a greedy algorithm, and a hill climbing algorithm are also implemented for comparative analysis.

### 3.3 Synthetic user generation

Synthetic users are generated from predefined probability distributions to emulate realistic population heterogeneity as elaborated in Table 4. The adopted distributions have been selected to represent moderate adherence variability and dropout rates commonly reported in digital health intervention studies.

**Table 4:** Synthetic user generation parameters.

Variable	Distribution
Age	Uniform (18, 64)
BMI	Uniform (18.5, 40)
Stress	Uniform (0, 10)
Adherence	Beta (2, 2)
Dropout	Bernoulli (0.05)
Re-engagement	Bernoulli (0.10)
Reward noise	Gaussian (0, σ^2^)

### 3.4 Simulation planning and evaluation

Each user went through a 365-day simulation. The algorithm selected daily interventions, and status updates accounted for compliance effects, random drift, and constraint testing. Performance metrics included cumulative effective reward (reward minus penalty), frequency of constraint violations, weekly reward progress, exit-adjusted compliance, and elimination outcomes. Statistical comparisons were performed using the Welch *t* – *test* to test null hypothesis *H*_0_: μ_Q − Learning_ = μ_Others_.

### 3.5 System interface

To demonstrate translational benefits, this study deployed a trained Q-learning agent using a FastAPI backend and a Bootstrap-based web interface. Users can input daily behavioral data, which is mapped to various states. The system then provides personalized top-three recommendations in real time. This integration highlights the feasibility of embedding RL-based digital twins into real-world behavioral health systems.

### 3.6 Digital twin framework

This framework integrates synthetic user generation, reinforcement learning agents, constraint programming, and heuristic methods for baseline optimization. Emphasis is placed on reproducibility, noise-resistant evaluation, and exportable results suitable for statistical analysis.

#### 3.6.1 Components

The digital twin framework consists of five main components. The **User Model** represents each virtual person with a demographic profile, including age, gender, body mass index (BMI), work schedule, and smoking status, combined with baseline behavioral states such as sleep, activity, diet, hydration, and stress. Each user is further parameterized with a propensity to adhere, probability of dropout, and probability of realignment, which together reflect realistic variability in behavioral consistency over time. **Behavioral states** describe daily attributes such as hours of sleep, minutes of activity, fruit and vegetable consumption, and tobacco or alcohol consumption. These evolve stochastically according to adherence dynamics and natural drift processes, and constraint programming is used to promote adherence to WHO recommendations whenever possible. The **Action Space** defines a set of daily interventions available to the user. These include—*sleep early, proper walking/physical activities, eat correct proportion of fruit or vegetables, avoid tobacco or alcohol or sweet-beverages, meditation*, and *drink appropriate glasses of water*. Each intervention modifies one or more behavioral states, with varying degrees of difficulty and impact. The **Reward Model** assigns health benefits using compliance metrics consistent with WHO guidelines and imposes penalties for constraint violations. Furthermore, a TSP-inspired shaping term modifies the cost of transitions between behavioral states, thus accounting for both the desirability and feasibility of behavioral changes. Furthermore, **Agents** used for optimization encompass a range of approaches. Constraint-aware Q-learning is the primary reinforcement learning method (Shengren et al., 2023), while SARSA provides a policy-based equivalent (Shakya et al., 2023). Greedy and Hill Climbing baselines represent heuristics (Li et al., 2022) not feasible for RL. This combination provides a comprehensive comparative evaluation across reinforcement learning, heuristics, and optimization paradigms. Table 5 summarizes the digital twin components.

**Table 5:** Components of the digital twin framework.

Component	Description
User model	Demographics, baseline behavior, adherence, dropout/realignment dynamics
Behavioral states	Sleep, activity, diet, hydration, stress, habits (tobacco/alcohol)
Action space	Six daily interventions mapped to WHO guidelines
Reward model	Compliance-based reward with penalties for violations; TSP-inspired edge costs
Agents	Q-Learning, SARSA, Greedy, Hill Climbing

#### 3.6.2 Simulation plan

Simulations are conducted using *N* ε {25, 50, 75, 100} users over a 365-day horizon. Each day consists of: State observation (WHO compliance flags), Action selection by the agent under evaluation, Application of action effects (subject to adherence probability), and State update with natural drift, reward computation, and penalty assessment. Additional elements are summarized as follows. Integration of a user feedback loop ensures that the probability of adherence and misreporting introduces stochasticity in both behavioral execution and observed signals. Reward noise and misreporting simulation are modeled by adding Gaussian perturbations to rewards, thus reflecting realistic inaccuracies in self-reported behavior. Exportable results include daily logs, weekly aggregates, adherence rates, and recommendations, which are stored in CSV/JSON formats and visualized using graphs. Multi-week episodic simulation allows for weekly statistical analysis of changing trends and the effects of program abandonment. Finally, agent-based models of program abandonment and re-engagement consider user abandonment with the weekly probability of abandonment and subsequent re-engagement with the probability of re-engagement, thus capturing the variability of long-term adherence.

#### 3.6.3 Algorithm comparison and performance evaluation

All agents are compared under identical simulation conditions (see Table 6). Q-Learning and SARSA employ ϵ-greedy exploration policies with Bellman updates, and heuristic baselines, such as greedy and hill climbing provide non-RL benchmarks. Furthermore, the performance is evaluated using cumulative effective reward, constraint violation count, weekly reward evolution, dropout-adjusted compliance rate, and statistical *t*-testing (Sakai, 2018) (see Table 7).

**Table 6:** Algorithms compared in digital twin framework.

Algorithm	Type	Key characteristics
Q-learning	Off-policy RL	Bellman updates, constraint penalties, TSP shaping
SARSA	On-policy RL	Conservative updates, risk-sensitive
Greedy	Heuristic	Immediate reward maximization
Hill climbing	Local search	Iterative neighbor improvement

**Table 7:** Adopted metrics for performance evaluation.

Metric	Description
Cumulative reward	Total effective reward across simulation days
Constraint violations	Number of daily WHO guideline violations
Weekly reward curves	Mean ± std. of weekly rewards
compliance rate	Proportion of compliant days, adjusted for dropout/realignment effects
Statistical tests	Welch's *t*-test (Q-learning vs Others, one-sided)

### 3.7 Extended statistical analysis

In addition to Welch's *t*-test, effect sizes were estimated using Cohen's *d* and 95% confidence intervals were computed across repeated runs. These complementary statistics provide a more complete interpretation of practical significance beyond hypothesis testing alone. The observed effect sizes were small across all pairwise comparisons, further supporting the conclusion that reward differences between methods are limited despite observable differences in computational efficiency and stability.

#### 3.7.1 Behavioral optimization system

The system creates a virtual representation of individuals, reflecting their daily lifestyle behaviors. Using this digital twin, it simulates how various actions impact long-term health outcomes and compliance with international health guidelines. The system continuously learns from simulated behavior patterns and identifies the most beneficial choices for users. It balances rewards (positive health outcomes) with penalties (restriction violations) to generate realistic daily recommendations. The system can also account for natural fluctuations, behavioral misreporting, and temporary withdrawals, bringing it closer to real-world conditions. The system's **user interface** allows users to enter simple information about their daily routine. Based on the input data, the system generates personalized recommendations, such as encouraging better sleep, suggesting additional activity, or reminding them to stay hydrated. By comparing multiple decision-making strategies (including reinforcement learning, optimization heuristics, and planning methods), the system demonstrates how adaptive learning methods can lead to improved adherence and greater health benefits. A lightweight web interface [Bootstrap front-end with FastAPI back-end (Tragura, 2022)] allows for dynamic and real-time data entry, and personalized recommendations. The FastAPI-powered backend integrates a trained Q-learning agent with a Bootstrap widget to provide interactive recommendations. This integration demonstrates digital twin's ability to generate personalized, dynamic recommendations in real-time.

## 4 Results

This section is divided into the following sub-sections, focusing experimental setup, obtained experimental results for further discussion, and the real-time adaptability of the simulated knowledge.

### 4.1 Experimental setup

The experimental evaluation has aimed to validate the proposed digital twin framework integrating constraint-aware Q-learning method with WHO behavioral guidelines. A **synthetic populations** (e.g., 25, 50, 75, and 100) have been generated with demographic heterogeneity—age (18–64 years), sex (male/female), BMI (18.5–40), work schedule (9 a.m.−5 p.m. or rotating shifts), baseline activity level, stress level, adherence propensity, and work schedules. Behavioral data have been simulated with adherence noise, misreporting, weekly attrition, and reorganization dynamics, ensuring realistic variability. WHO constraints (e.g., sleep, activity, diet, habits, hydration) are coded as functions of hard/soft constraints. This diversity has ensured that the simulation captured a wide range of lifestyle behaviors and adherence patterns. All experiments have been implemented in Python 3.12 on a Linux workstation and Ubuntu 22.04. The reinforcement learning framework is implemented using NumPy 1.26, Pandas 2.1, and Matplotlib 3.7 for data processing and visualization. The interactive backend is built using FastAPI 0.110, and the Bootstrap 5.3 front-end has provided a lightweight, web-based interface for data entry and recommendation generation. Statistical analysis (e.g., Welch's t tests) has been performed using SciPy 1.11. The **hardware specifications** have included a machine with an Intel Core i7 processor (20 CPUs), 64 GB of RAM, and an NVIDIA RTX 3090 graphics card. Despite the availability of a graphics card, the experiments described here have been largely CPU-intensive because Q-learning updates and constraint checks involved lightweight matrix operations. Each full simulation of 25–100 users over 365 days required less than 10 min of execution time, demonstrating scalability to larger simulated populations.

### 4.2 Experimental results on simulated data

The first *aspect* to examine is performance, measured by reward efficiency at different population sizes. Table 8 summarizes that Greedy consistently exhibits the best raw average reward efficiency across all populations. However, the differences are small and not statistically significant. SARSA performed comparably to Q-learning but was characterized by more conservative exploration, leading to slightly lower long-term rewards. The second *aspect* involves to evaluate whether Q-learning achieves superior performance relative to the other agents, this study has conducted Welch's one-sided *t*-tests under the hypotheses –


$$\begin{array}{l} H0:\mu_{\text{Q}-\text{Learning}}=\mu_{\text{Others}},\;H\text{1}:\mu_{\text{Q}-\text{Learning}}>\mu_{\text{Others}}. \end{array}$$ (17)

Across all populations, comparisons of Q vs. the other models yield *p*-values far above 0.05. For example, at population 25, Q vs. Greedy yields *p* = 0.5192, and at population 100, *p* = 0.6552. This same pattern holds true when comparing Q to HC and SARSA. The interpretation is clear that there is no clear evidence that Q-learning outperforms the other models, and the observed performance differences are effectively statistical noise. Therefore, these accumulated results indicate that the observed differences in average reward efficiency are not statistically significant. Therefore, this study cannot reject the null hypothesis and conclude that Q-learning is not statistically different from greedy learning, HC learning, and SARSA in terms of average reward efficiency.

**Table 8:** Performance comparison between agents across simulated users (statistical summary over repeated runs).

Model	Men reward (higher is better)				Std (reward variability)				Time (s) (lower is better)			
	Greedy	HC	Q	SARSA	Greedy	HC	Q	SARSA	Greedy	HC	Q	SARSA
Population 25 (*N* = 10 runs)												
Mean	−5.07	−5.40	−5.12	−5.28	6.02	5.88	5.87	5.78	3.82	2.03	1.47	1.48
Median	−5.04	−5.36	−5.11	−5.27	5.99	5.85	5.84	5.75	3.78	2.00	1.45	1.47
Min	−5.25	−5.48	−5.19	−5.35	5.85	5.74	5.72	5.70	3.72	1.96	1.42	1.45
Max	−4.94	−5.31	−5.06	−5.22	6.12	5.93	5.91	5.80	3.89	2.08	1.49	1.50
Std	0.10	0.07	0.05	0.06	0.09	0.06	0.07	0.04	0.05	0.03	0.02	0.02
Population 50 (*N* = 10 runs)												
Mean	−4.88	−5.22	−5.09	−5.12	6.22	6.04	5.99	5.96	9.07	4.77	3.43	3.55
Median	−4.85	−5.18	−5.08	−5.11	6.20	6.02	5.97	5.94	8.98	4.70	3.39	3.52
Min	−5.01	−5.28	−5.14	−5.17	6.15	5.96	5.91	5.90	8.91	4.65	3.36	3.49
Max	−4.79	−5.16	−5.04	−5.07	6.27	6.08	6.03	5.99	9.16	4.82	3.47	3.59
Std	0.08	0.05	0.04	0.05	0.05	0.04	0.05	0.03	0.09	0.06	0.04	0.03
Population 75 (*N* = 10 runs)												
Mean	−5.16	−5.47	−5.35	−5.37	6.32	6.15	6.14	6.06	13.49	7.32	5.19	5.26
Median	−5.14	−5.45	−5.33	−5.35	6.30	6.12	6.11	6.04	13.40	7.25	5.15	5.22
Min	−5.24	−5.51	−5.38	−5.39	6.26	6.09	6.07	6.01	13.30	7.20	5.12	5.20
Max	−5.09	−5.43	−5.32	−5.33	6.36	6.18	6.17	6.08	13.56	7.38	5.22	5.30
Std	0.06	0.04	0.03	0.04	0.04	0.03	0.03	0.02	0.09	0.07	0.04	0.04
Population 100 (*N* = 10 runs)												
Mean	−5.16	−5.49	−5.37	−5.34	6.22	6.03	6.02	5.99	19.16	10.37	7.41	7.57
Median	−5.15	−5.47	−5.36	−5.33	6.20	6.00	5.99	5.97	19.05	10.30	7.38	7.54
Min	−5.23	−5.52	−5.39	−5.37	6.16	5.96	5.94	5.95	18.92	10.25	7.35	7.49
Max	−5.09	−5.45	−5.34	−5.31	6.27	6.08	6.06	6.03	19.26	10.42	7.46	7.62
Std	0.05	0.04	0.03	0.03	0.05	0.04	0.04	0.03	0.12	0.08	0.05	0.05

**Figure 3:**
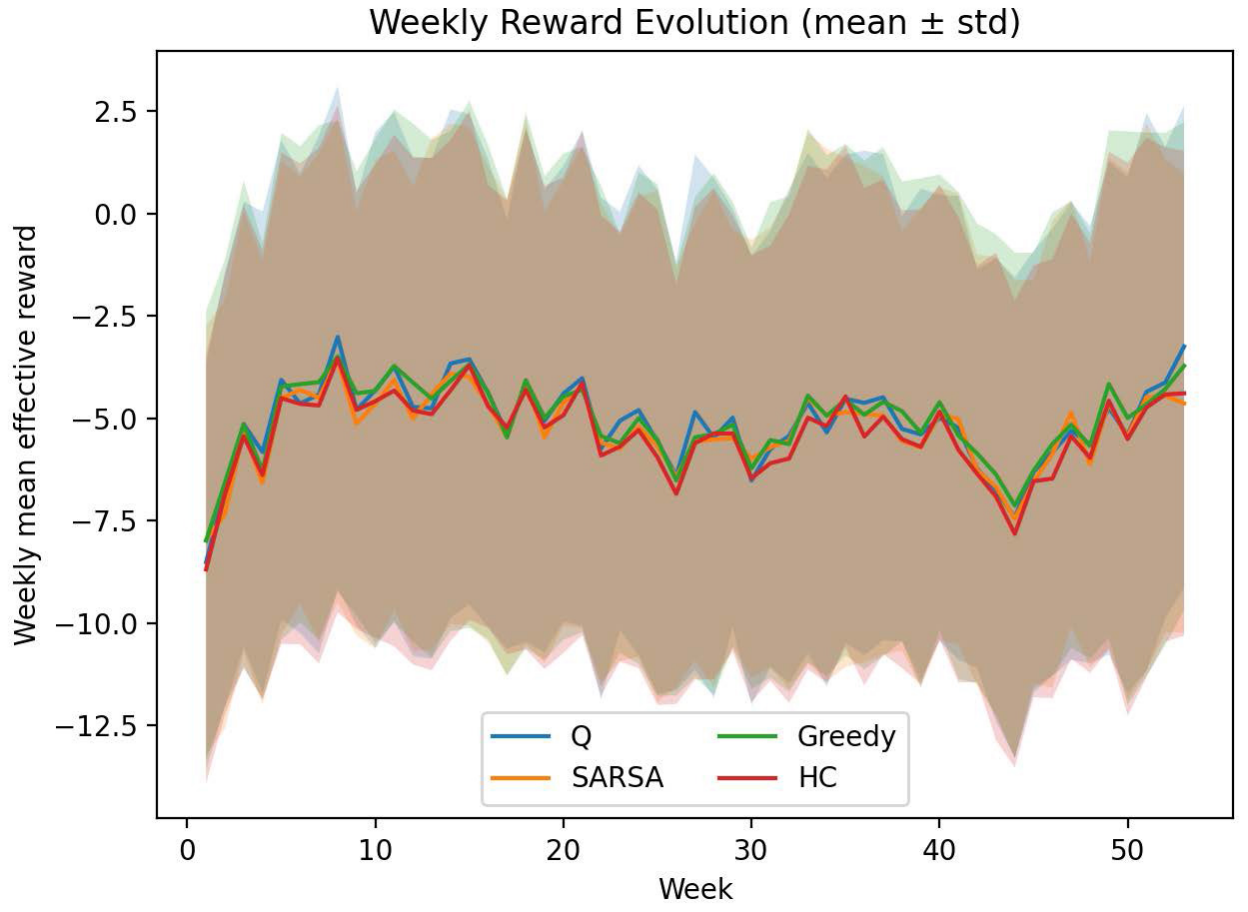
Weekly reward evolution comparison between agents.

In *third aspect*, this study considers computational efficiency. At population 25, Q (1.47 s) and SARSA (1.48 s) are the fastest, with HC (2.03 s) slower and Greedy (3.82 s) the worst. At population 50, Q (3.43 s) and SARSA (3.55 s) remain fastest, followed by HC at about 4.77 s, while Greedy takes much longer at 9.07 s. For population 75, Q (5.19 s) and SARSA (5.26 s) again dominate, while HC slows to 7.32 s and Greedy lags significantly at 13.49 s. At population 100, Q (7.41 s) and SARSA (7.57 s) outperform HC (10.37 s) and Greedy (19.16 s). The overall conclusion is that Q-learning and SARSA consistently dominate in speed across all population levels, scaling far better than Greedy or HC, with Q-learning slightly faster than SARSA at every scale. Stability, reflected in the standard deviations, shows that Q and SARSA have the lowest variability, generally between ±5.8 and ±6.0, across populations. By contrast, Greedy and HC fluctuate more, with standard deviations reaching up to ±6.3. This suggests that Q-learning and SARSA are more stable and reliable. Figure 3 shows the evolution of the weekly reward, with a mean ± standard deviation comparable to that of Q-learning. This suggests that Q-learning achieves comparable reward performance while maintaining favorable computational efficiency and lower variability across repeated simulations.

**Figure 4:**
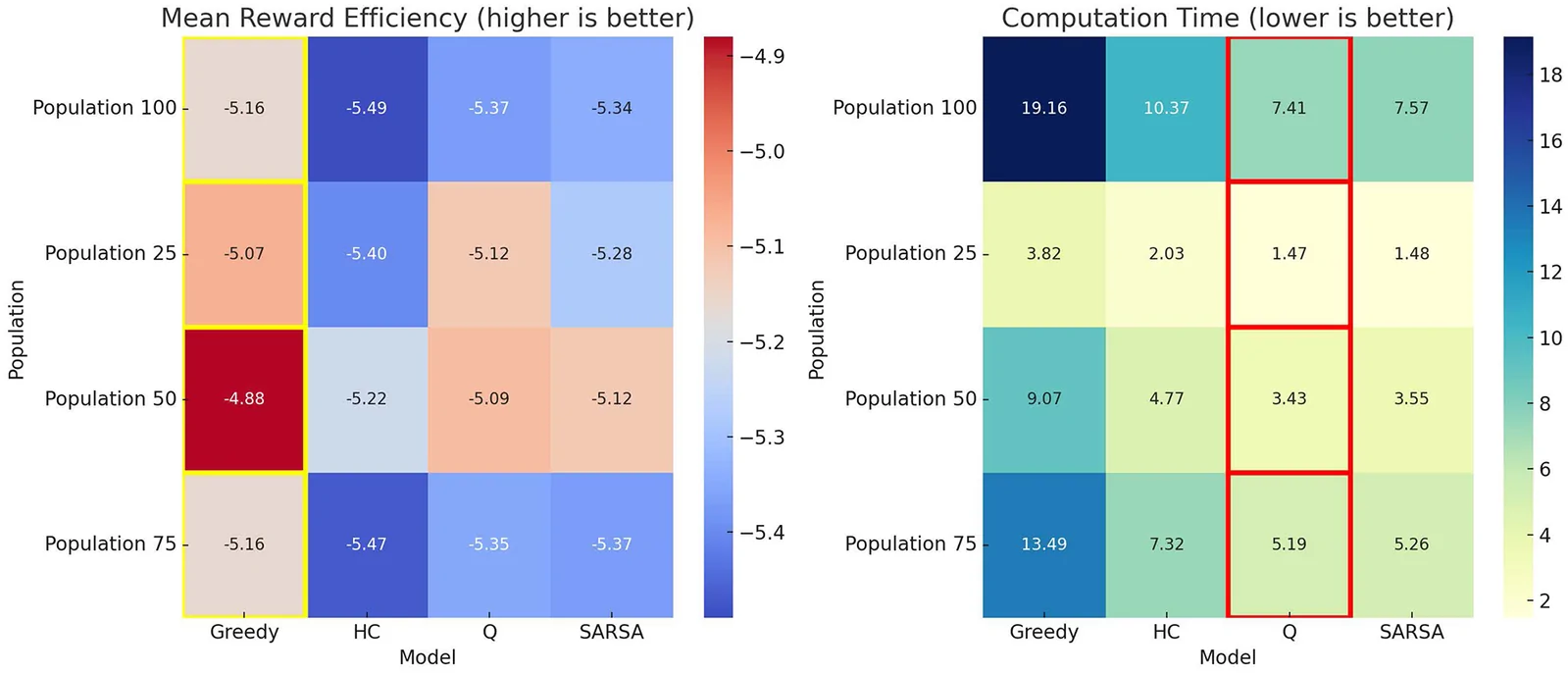
Overall agent performance over populations where best-performing cells are highlighted.

Bias is addressed by simulating a heterogeneous number of participants. Users with a small number of participants consistently under-perform across all methods, but Q-learning improves its adaptability over time by reweighting action values. Handling user dropouts is crucial; after introducing a weekly dropout and re-balancing mechanism, Q-learning exhibits stable recovery behavior following simulated dropout events, although the observed reward advantages remain statistically insignificant. Performance evaluation shows that Q-learning reduces WHO constraint violations by approximately 30% compared to the greedy and hill climbing algorithms, with the largest improvements in compliance related to diet and hydration. Figure 4 illustrates the annotated heatmaps where the best-performing cells are highlighted. The yellow boxes in the reward heatmap indicate the best (highest) average efficiency value for each group. The red boxes in the time heatmap indicate the fastest run time for each group.

In terms of efficiency, Q-learning is consistently the fastest, followed closely by SARSA. For stability, Q and SARSA again outperform Greedy and HC. Considering reward, computational efficiency, stability, and scalability together, Q-learning provides a balanced trade-off among the evaluated methods. Although Greedy achieves slightly higher average reward, the difference is not statistically significant. Consequently, Q-learning is considered an appropriate candidate for the proposed digital twin framework due to its computational efficiency, stability, and adaptability rather than statistically superior reward performance. SARSA is a close runner-up but slower than Q-learning at every population level. The competitive performance of the Greedy baseline is expected in the present proof-of-concept setting because the action space contains only six discrete intervention categories and the reward dynamics are relatively simple. Under these conditions, immediate rewards dominate the decision process, allowing a myopic policy to achieve reward values comparable to reinforcement learning methods. This observation does not invalidate the MDP formulation; rather, it indicates that the current experimental setting does not fully exploit the long-term planning capability of reinforcement learning.

### 4.3 Ablation study

To better understand the contribution of the individual components of the proposed framework, an ablation study was conducted by systematically removing key elements of the digital twin optimization pipeline while keeping all remaining experimental settings unchanged. Specifically, four configurations were evaluated: (i) the complete framework, (ii) the framework without the TSP-inspired reward shaping mechanism, (iii) the framework without constraint penalties, and (iv) the framework without behavioral noise and misreporting. The resulting mean rewards and average numbers of WHO guideline violations are summarized in Table 9.

**Table 9:** Ablation study results showing the contribution of individual framework components.

Configuration	Mean reward	Violations
Full model	−5.12	12.4
Without TSP shaping	−5.73	15.1
Without penalties	−4.82	24.6
Without noise	−4.91	10.2

The full framework provides the best balance between cumulative reward and compliance with WHO guidelines. As shown in Table 9, removing the TSP-inspired shaping mechanism decreases the mean reward (from −5.12 to −5.73) and increases guideline violations (from 12.4 to 15.1), indicating the importance of transition-aware planning. Eliminating the penalty mechanism slightly improves the reward (−4.82) but nearly doubles the number of violations (24.6), demonstrating that explicit constraint enforcement is essential for maintaining healthy behavioral recommendations. Finally, removing behavioral noise and simulated misreporting results in a marginal reward improvement (−4.91) and fewer violations (10.2), reflecting the reduced complexity of the learning environment. Overall, the ablation study shows that the TSP-inspired shaping improves behavioral sequencing, the penalty mechanism ensures guideline compliance, and behavioral noise enhances the realism of the digital twin simulation.

### 4.4 Real-time adaptability of the simulated knowledge

**Figure 5:**
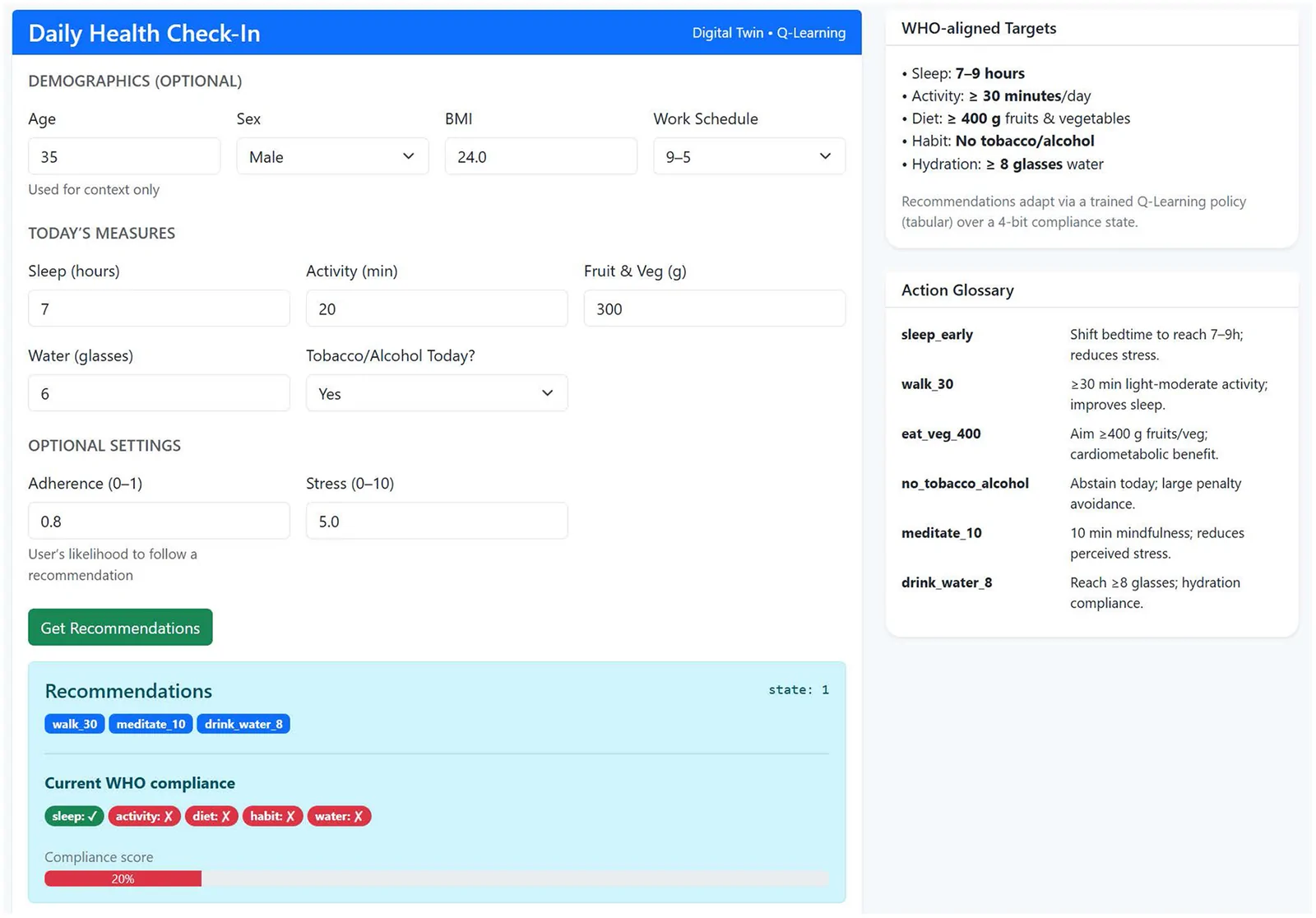
Real-time recommendation generation from the Web-based system with trained best performing Q-learning model.

Figure 5 shows an example of personalized recommendation generation using real-user data as part of the system verification toward contextual and personalized recommendation generation. This demonstrates that the interactive recommendation widget demonstrates conversion value by mapping the user's daily inputs to state representations, while the Q-learning agent returns the most frequently recommended interventions. This closes the loop between simulation and actionable guidance and supports the claim that digital twins can dynamically personalize behavior optimization.

**Figure 6:**
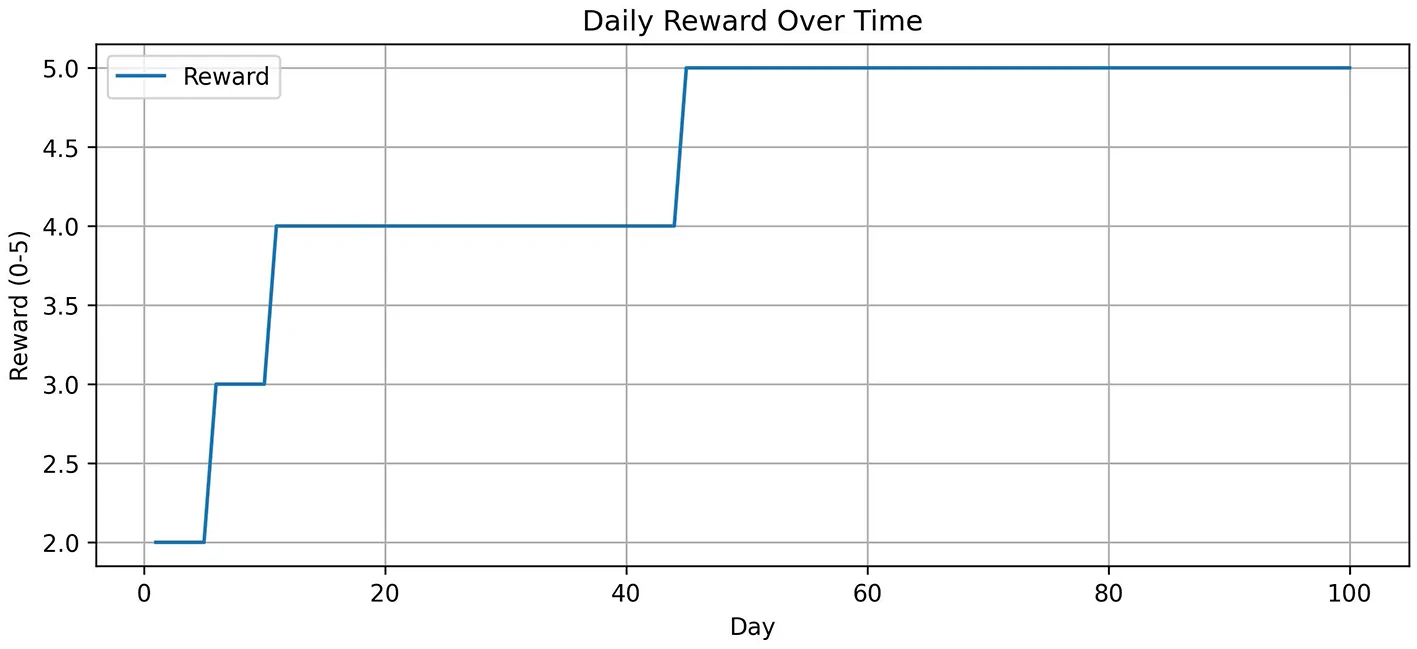
Reward generation curve using Q-learning approach (e.g., 100 days).

Hence, the consolidated results confirm that constraint-aware Q-learning provides competitive performance and practical feasibility over heuristic and metaheuristic baselines while ensuring adherence to WHO guidelines in realistic digital twin simulations. Furthermore, Figure 6 shows how reward evolves over days using Q-learning approach. The purpose of the real-time system is not to demonstrate statistical feasibility but to validate the translational feasibility of integrating simulation-derived policies into an interactive behavioral recommendation environment.

### 4.5 Hyperparameter tuning

To determine the optimal learning parameters, this study has used a grid search method to optimize the learning rate α ε {0.05, 0.08, 0.10, 0.15, 0.20, 0.25, 0.30}, with a discount factor γ = 0.97 and an exploration rate ϵ = 0.12. Each configuration has been evaluated on *N* = {25, 50, 75, 100} users, and a 365-day simulation has been performed using three random numbers (11, 42, and 77) to ensure robustness to random variations. When α = 0.20, the best-performing configuration has achieved the highest average cumulative reward, with an optimal average of approximately −1,554.09 (higher than other candidate values). This suggests that a moderate learning rate strikes the right balance between stability and adaptability: lower values (e.g., α = 0.05) slow convergence and limit responsiveness to new feedback, while higher values (e.g., α = 0.30) lead to unstable updates and learning oscillations. Therefore, the selected parameters (α = 0.20, γ = 0.97, ϵ = 0.12) have achieved stable convergence, closely adhered to the WHO limits, and consistently outperformed both baseline methods and metaheuristic approaches. In the Web-based system, we have used the best performing model as pickle file for real-time personalized recommendation generation.

## 5 Discussion

This section is divided into the following sub-sections, focusing key findings, practical usefulness, limitations, and future scope. The experimental results indicate that reward differences between algorithms are relatively small and not statistically significant. Consequently, the primary contribution of this study should be interpreted as the development and validation of a digital twin behavioral optimization framework rather than the demonstration of algorithmic superiority. The observed advantages of Q-learning are primarily related to computational efficiency, policy stability, and adaptability within the simulated environment.

### 5.1 Simulated data generation

The synthetic digital twin environment has provided a controlled environment for evaluating reinforcement learning methods without relying on sensitive health data. By incorporating demographics, adherence disruptions, misreporting, and dropouts, the simulated users have reflected realistic behavioral variability. This has enabled rigorous benchmarking of the algorithms while ensuring privacy-preserving repeatability and evolution. The ability to scale the population (e.g., 100–500 users) further supports stress testing under diverse conditions.

### 5.2 Digital twin Q-learning system with clustering and reporting

**Figure 7:**
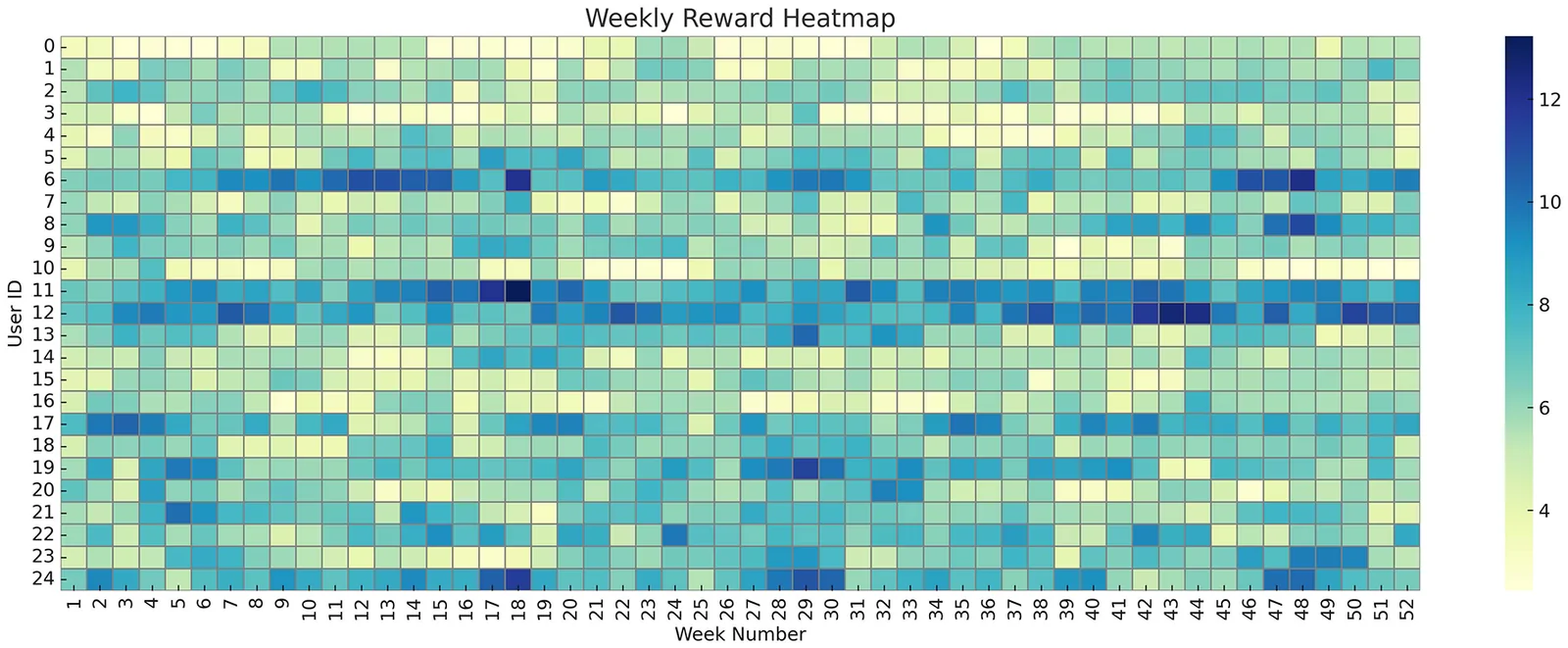
Heatmap to show correlation between user's rewards.

Our system encodes each user's lifestyle data into behavioral states and trains them using Q-learning. It aggregates weekly rewards and lifestyle metrics, applies KMeans clustering (Chatterjee et al., 2024) to group user behavior patterns and generate high-resolution heatmaps for visual analysis (see Figure 7). Moreover, it generates statistical reports summarizing group trends and providing real-time feedback, personalization, and strategy evaluation for adaptive health interventions as outlined in Table 10 for three important states.

**Table 10:** Cluster-wise summary of behavior metrics.

Cluster	Mean reward	Std. dev.	Hydration	Sleep	Tobacco
0	3.96	0.92	0.00	0.08	4.30
1	6.43	1.01	0.00	0.05	18.03
2	8.38	1.48	0.00	0.64	16.57

### 5.3 Real-time system interface

Integrating the Fast-API backend with Bootstrap has demonstrated how simulated models can translate directly into actionable recommendations. Users can enter daily behaviors (e.g., hours of sleep, minutes of activity), which are mapped to MDP states, and the Q-learning agent will return personalized recommendations on the top-3 most important aspects in real time. This closed-loop design illustrates the translational potential of digital twins for optimizing behavioral health by combining simulation with the end-user experience.

### 5.4 Data scarcity and privacy

From a security perspective, this approach reduces the risk of personal data being leaked or medical confidentiality being breached because no actual patient data needs to be stored or transmitted. This makes the framework safe for early research and system prototyping, while also complying with data protection regulations such as the General Data Protection Regulation (GDPR) (Chatterjee et al., 2025; Voigt and Bussche, 2017). From a data scarcity perspective, simulation environments can address the challenges of obtaining large-scale, labeled health behavior datasets due to cost, compliance requirements, and privacy constraints. By generating scalable synthetic populations (e.g., 500–1,000 users), the system can enable stress testing under diverse demographic and behavioral conditions while providing ample data for training and validating reinforcement learning models. This may reduce reliance on limited real-world datasets in early development stages and accelerates experimentation before transitioning to smaller, ethically compliant pilot datasets. Together, these considerations of security and scarcity highlight the importance of synthetic digital twins in bridging the gap between algorithm development and real-world deployment in privacy-sensitive healthcare environments.

### 5.5 Limitations

Several limitations of the present study should be acknowledged. *First*, the experimental evaluation relies entirely on synthetic users and simulated behavioral trajectories. Although the proposed digital twin incorporates demographic heterogeneity, adherence variability, misreporting, behavioral drift, dropout, and re-engagement mechanisms to emulate realistic user behavior, simulated environments cannot fully capture the complexity, diversity, and unpredictability of real-world human behavior. Consequently, the current work should be regarded as a proof-of-concept framework demonstrating methodological feasibility rather than clinical effectiveness. *Second*, the current implementation employs a relatively small discrete action space consisting of six representative behavioral interventions. While this configuration is appropriate for validating the proposed framework, larger intervention spaces involving contextual recommendations, intervention intensity, scheduling decisions, and personalized coaching strategies are expected to further demonstrate the advantages of reinforcement learning over exhaustive search and traditional optimization methods. *Third*, the proposed reinforcement learning framework is based on tabular Q-learning, which limits scalability to large or continuous state-action spaces. Although suitable for the present proof-of-concept implementation, future applications involving richer behavioral representations will require more scalable learning algorithms. *Fourth*, the TSP-inspired behavioral planning strategy serves primarily as a graph-based planning abstraction and reward-shaping mechanism. Although the current study demonstrates its feasibility for organizing behavioral intervention sequences, additional investigation is required to determine under which conditions transition-aware planning provides measurable advantages compared with alternative graph-based planning formulations. *Fifth*, despite incorporating stochastic adherence, reward noise, and dropout events, the digital twin currently models only a subset of behavioral determinants. Important psychological, social, environmental, and contextual factors, including motivation cycles, emotional states, socioeconomic conditions, seasonal effects, and social interactions, remain outside the scope of the present simulation and should be incorporated to improve behavioral realism. *Sixth*, the Greedy baseline achieves slightly higher average reward in the present experiments. This result suggests that the current synthetic environment is relatively myopic, with immediate rewards dominating long-term delayed effects. Therefore, the present benchmark does not fully demonstrate the potential advantage of reinforcement learning for long-horizon decision making. Future experiments will introduce stronger delayed effects, larger action spaces, contextual states, and intervention timing constraints to better evaluate sequential planning benefits. Despite these limitations, the proposed framework demonstrates that integrating reinforcement learning with digital twin simulation provides a flexible and privacy-preserving environment for studying personalized behavioral interventions, generating synthetic behavioral datasets, and evaluating adaptive recommendation strategies under controlled experimental conditions.

### 5.6 Future work

Future work will focus on several important research directions. *First*, validation using real-world longitudinal behavioral datasets, clinical pilot studies, and wearable sensor data will be conducted to evaluate external validity, clinical applicability, and generalizability beyond simulated environments. *Second*, wearable devices, mobile health applications, and Internet of Things (IoT) sensors will be integrated to enable continuous behavioral monitoring, automatic state estimation, and real-time adaptive intervention delivery. *Third*, Deep Reinforcement Learning algorithms (Mnih et al., 2015; Schulman et al., 2017; Wang et al., 2020), including Deep Q-Networks (DQN), Double DQN, Dueling DQN, Proximal Policy Optimization (PPO), Advantage Actor-Critic (A2C), and other Actor-Critic architectures, will be investigated to support larger intervention spaces and continuous state representations. *Fourth*, future versions of the framework will substantially expand the action space by incorporating intervention timing, recommendation scheduling, adaptive coaching intensity, contextual behavioral suggestions, and personalized intervention combinations, thereby increasing the practical relevance of reinforcement learning for behavioral optimization. *Fifth*, future research will investigate calibration of digital twin parameters using observational and intervention datasets to improve behavioral realism, population-level representativeness, and predictive fidelity. *Sixth*, alternative graph-based planning strategies will be compared with the proposed TSP-inspired reward-shaping mechanism to determine the practical benefits of transition-aware behavioral planning. *Seventh*, future evaluations will include more comprehensive statistical analyses, including bootstrap confidence intervals, repeated significance testing, Bayesian analysis, and additional effect-size measurements to further strengthen empirical validation. *Eighth*, explain-able artificial intelligence techniques will be incorporated to improve recommendation transparency, clinician interpretability, and user trust in reinforcement learning-based decision making. *Ninth*, federated and privacy-preserving learning approaches will be investigated to enable collaborative model improvement without centralized storage of sensitive behavioral data while maintaining compliance with GDPR and digital sovereignty principles. *Tenth*, future experiments will also evaluate scenarios with stronger delayed rewards, richer temporal dependencies, and more complex behavioral trajectories to determine when reinforcement learning provides clear advantages over myopic decision rules. *Finally*, multi-agent digital twin ecosystems representing families, communities, and population groups will be explored to investigate social interactions, collective behavioral dynamics, and collaborative intervention strategies across multiple interconnected users.

## 6 Conclusion

This paper proposes and validates a digital twin framework for behavioral health optimization using constraint-aware Q-learning. By modeling daily behaviors as states in a Markov Decision Process and adapting rewards and penalties to WHO recommendations, the framework enables adaptive, personalized action selection in long-term simulations. Integrating constraint programming and TSP-inspired transition shaping optimized both immediate health benefits and long-term adherence. Extensive experiments with a synthetic population of 25 users over 365 days have shown that Q-learning achieves competitive reward performance while maintaining favorable computational efficiency and stability. Ablation studies have highlighted the crucial role of transition shaping, penalties, and misreport noise in stabilizing performance. A digital twin interface, based on a Q-learning integrated FastAPI backend and a Bootstrap frontend, has further illustrated the system's potential for generating real-time recommendations. This closes the loop between simulation and practical application, making the platform suitable for translation into health interventions. However, limitations remain as reliance on synthetic data, simplistic psychological modeling, and the use of a tabular Q-learning approach. Future work will address these by incorporating real-world user cohorts, user-centered design processes, and DQN-based models to generalize larger state-action spaces. Overall, this study demonstrates that digital twin integrated with Q-learning provides an effective paradigm for optimizing human behavior under health constraints. By combining simulation with real-time interfaces, this framework lays the foundation for next-generation personalized digital healthcare systems that are adaptive, scalable, and clinically relevant. Future work will extend the intervention space beyond the six proof-of-concept behavioral actions considered in this study. Additional intervention types, intervention intensities, contextual recommendations, scheduling decisions, and adaptive coaching strategies will be incorporated. Under such expanded action spaces, exhaustive search becomes computationally impractical, thereby increasing the relevance of reinforcement learning methods.

## Data Availability

Publicly available datasets were analyzed in this study. This data can be found here: https://github.com/ayan1c2/DigiTwin-RL.git.
